# Mediators of Inflammation in Pulmonary Diseases

**DOI:** 10.1155/2015/739219

**Published:** 2015-08-10

**Authors:** Kostas Spiropoulos, Nikolaos Siafakas, Marc Miravitlles, Francesco Blasi, Kiriakos Karkoulias

**Affiliations:** ^1^Department of Pulmonary Medicine, University Hospital of Patras, University of Patras, 26500 Patras, Greece; ^2^Department of Thoracic Medicine, University General Hospital, Medical School, University of Crete, 71110 Heraklion, Greece; ^3^Department of Pulmonary Medicine, Hospital Universitari Vall d'Hebron, 08036 Barcelona, Spain; ^4^Department of Respiratory Medicine, Università degli Studi di Milano, 20122 Milan, Italy

Inflammation is supposed to play a great role in the pathogenesis of the most common diseases of the respiratory system. In Chronic Obstructive Pulmonary Disease (COPD), which is a major cause of morbidity and mortality all around the world, smoking mainly causes the initiation of the inflammatory process that leads to an impaired respiratory function. Moreover, in bronchial asthma there are numerous proinflammatory mediators that are responsible for the onset and the progression of the disease. Finally, pulmonary infections and especially tuberculosis result in the orchestration of an inflammatory process that targets the causative agent in order to protect the host.

L. M. O. Caram et al. have evaluated the levels of vitamin A in the serum and sputum and attempted to correlate it with known inflammatory markers, such as tumor necrosis factor alpha (TNF-*α*), interleukin- (IL-) 6, IL-8, and C-reactive protein (CRP) in 50 COPD patients and 50 individuals without COPD. The authors concluded that serum concentration of vitamin A is negatively associated with the presence of COPD and that it is positively associated with smoking status. Although COPD patients exhibited increased inflammation, these inflammatory markers were not associated with serum retinol concentrations.

The manuscript of W. Zhang et al. tested the effect of a novel *γ*-secretase inhibitor to promote Th17 cell differentiation in a mouse model of allergic asthma. Their findings suggest that the inhibitor directly regulates Th17 responses in the mouse model of allergic asthma that they used, making it potentially efficacious.

In their study, R. Rajajendram et al. tried a synthetic chalcone analogue in a murine model of asthma. Their results demonstrate a potential role of the substance in asthma, as the treatment with it inhibited eosinophilia, goblet cell hyperplasia, peripheral blood total IgE, and airway hyperresponsiveness in ovalbumin-sensitized and challenged mice. However, the tested nonsteroid potentially anti-inflammatory substance is far away from clinical use.

In the manuscript entitled “Binding of CXCL8/IL-8 to* Mycobacterium tuberculosis* Modulates the Innate Immune Response,” A. Krupa et al. illustrate the role of IL-8 in the pathogenesis of tuberculosis (TB). The authors investigated the contribution of IL-8 in the inflammatory processes that are typically elicited in patients with TB. Their findings show that IL-8 seems to be the major chemokine responsible for recruiting T lymphocytes (CD3+, CD4+, and CD8+ T cells) and plays important role in the innate immunity against* Mycobacterium tuberculosis*.

Last but not least, the study of R. Baumann et al. investigated the IgA and IgG responses to mycobacterial protein antigens in subjects with latent tuberculosis, active tuberculosis, and healthy individuals. In their conclusions they present a new biomarker for the diagnosis of active pulmonary tuberculosis.

